# Special Issue with Research Topics on “Recent Analysis and Applications of Mass Spectra on Biochemistry”

**DOI:** 10.3390/ijms25041995

**Published:** 2024-02-07

**Authors:** Bojidarka Ivanova

**Affiliations:** Independent Researcher, 44139 Dortmund, Germany; bojidarka.ivanova@yahoo.com or b_ivanova@web.de

Analytical mass spectrometry applies irreplaceable mass spectrometric (MS) methods to analytical chemistry and chemical analysis, among other areas of analytical science. There are ongoing debates [1,2,3] on the definitions of analytical chemistry. These definitions by Zolotov (2021) [1] and Adams and Adriaens (2021) [3], labeled (1) and (2) below, respectively, shaped the research tasks of the latter field:

(1) “Analytical Chemistry is the science creating and developing the general methodology, methods, and means of the determination of the chemical composition and chemical structure of substances and developing methods of chemical analysis of particular material samples [1]”.

(2) “Analytical chemistry is the autonomous and fundamental scientific field involved with the development of methods for the complete or partial compositional and structural description in space and/or time of specific, natural, or man-made material objects or representative portions thereof, in order to relate this information with properties or functional or other characteristics of the objects. While its name historically refers to its origins in chemistry, analytical chemistry now applies any chemical, physical, biological, or other principles and methods to pursue its specific objectives. The discipline feeds and is connected to chemical analysis, a related applied scientific field, which is involved with various applications of analytical chemistry for either fundamental research in various scientific disciplines, or for technological or societal applications [3]”.

Therefore, analytical chemistry uses measurands of chemical analysis, thus detailing the analyte amount, its molecular properties, 3D molecular conformation, and electronic structure via instrumental methods. From the perspective of the themes of the Special Issue, analytical mass spectrometry uses measurable variables of the mass spectrum of chemicals. Methodologically, the field elaborates not only MS instrumentation and techniques but also methods for the data processing of measurands. The latter approaches are implemented into so-called omics methods, thus gaining crucial knowledge of biological systems. Fields of bioinformatics utilize these bioanalytical tools [3,4,5]. 

Among the various MS methods, soft ionization methods have become the gold standard in analytical practice [6,7]. They exhibit superior instrumental features and performances, thus showing (a) ultra-high accuracy, precision, reproducibility, sensitivity, reliability, selectivity, and specificity; (ii) capability of low- and high-molecular weight analyte (10–100 kDa) analyses; (iii) low concentration limits chemical of detection and quantitation within the framework of attomole to fmol levels [8,9]; and (iv) ultra-high resolving power, determining the error of mass-to-charge (*m*/*z*) measurement ~1 ppm [10,11,12,13], respectively. These performances are achieved via high-resolution mass analyzers, in particular Orbitrap [11,12,13,14] and Fourier transform (FT) ion cyclotron resonance (ICR) [14,15,16,17]. The former analyzer was developed by Makarov [18], while the latter was developed by Comisarow and Marshall, respectively [19]. The orbital ion trapping phenomenon established by Kingdon [20] is also used in designing the Orbitrap analyzer. These industrial-scale implemented innovations have resulted in crucial methodological developments in analytical mass spectrometric instrumentation [2,14,21,22,23,24], respectively, in a large number of multidisciplinary research fields utilizing mass spectrometric approaches. 

The dynamically harmonized measurement cell of FTICR-MS, designed by Nikolaev and Boldin [25,26], refines isotopomers of peptides and proteins [27]. The newcomer solves problems with the FT-ICR-MS phenomena of inhomogeneity of trapping electric fields [28] and ion coalescence [29] because FTICR-MS is often limited by space-charge effects, thus shifting and broadening MS peaks. There is systematic spectral error in proteomics and metabolomics due to the merging of two close MS peaks in FT-ICR-MS experiments. Explanatory and predictive theories of these phenomena have been developed, respectively, by Boldin and Nikolaev [29] and Naito and Inoue [30,31]. There is a decrease in the error contribution to MS measurands from ion sources, analyzers, and detectors, thus obtaining the mass spectrum of the analyte and exhibiting its fine isotope distribution. The task is challenging even when utilizing the latest generation of FT-ICR or Orbitrap analyzers. There is also theory detailing the space charge shift of ICR frequency by Jeffries and co-workers [32]. The same phenomenon has also been described theoretically by Gorshkov, Marshall, and Nikolaev [33]. The molecular isotopologies yield a relative isotopic abundance of stable natural isotopes of atoms in analytes, thus increasing the crucial reliability of their identification and annotation. The task is important for omics protocols used in clinical precision medicine due to the compulsory request for high analytical standard method performance of omics analyses. 

However, so-called fluctuations in elemental composition are due to isotope fractionation obtained by (bio)chemical and geochemical processes [27]. They perturb the value of the isotope ratio among isotopes of the same atom and also yield errors in proteomics [34] or isotomics [27,35]. Highly precise measurands of isotopomers contribute crucially not only to the fields of medicine and clinical diagnostics but also to ecology, geology, history, forensic anthropology, and more [36]. 

In highlighting the ultra-high resolution of MS measurands, it should be mentioned that the already achieved single-sample analysis of 126,264 species using 9.4T FT-ICR-MS, in addition to the highest broadband accuracy and resolving power obtained via MS methods at 21 T [16,17,37], is 3.105 resolution at *m*/*z* 400. A resolving power of 2.106 has been detected when studying proteins over a measurement span time t = 12 s. The Orbitrap analyzer accurately determines the *m*/*z* value of heterogeneous viral specials and oligomers of immunoglobulin with high charges [38,39]. 

Mass spectrometric methods also exhibit (v) (automated) direct analysis and assay without employment in sample pre-treatment [40,41,42,43,44]; (vi) flexible (and portable) instrumentation coupled to methods of chromatography, electrochemistry, and more; (vii) lab-on-chip technologies; and (viii) miniaturized instrumentation devices and techniques [45,46], respectively. A miniature mass spectrometer achieves fast monitoring (*t*~4 min) of therapeutics in a whole blood sample (*r*^2^ = 0.9962) [46,47]. The methods (ix) adopt imaging techniques [27]. The so-called imaging mass spectrometry assesses living cells, organs, microorganisms [48,49], and whole bodies [50,51,52,53,54,55,56,57], as well as determines, free of isotope labeling, hundreds to thousands of chemicals, metabolites, lipids, proteins, and more in tissue within the framework of a single experiment [50]. Applications of the technique to the biochemistry of lipids in tissues have been highlighted comprehensively in the review article [53]. Monitoring of bacterial growth has been illustrated (2022) [58].

Mass spectrometric approaches (x) monitor continuous flow chemical reactions; (xi) examine complex analyte mixtures in biological tissues and fluids [37,40,59], environmental [60,61,62], and foodstuff samples; (xii) are used for in vivo diagnostics; and (xiii) experimentally determine kinetics, thermodynamics, diffusion, and ion mobility parameters of chemicals and their reactions [63,64,65,66,67,68]. 

The kinetic method developed by Cooks and co-workers shows many advantages, among others [69]. In addition, a linear correlation between the energetics of MS reactions based on Hammett free energy and kinetic parameters has been established by McLafferty and co-workers [70]. Data on the intensity of peaks of the mass spectra of parent and product ions of analytes examining reaction kinetics show a linear relation between intensity peak ratio and time of chemical reaction (*r*^2^ = 0.99) [64,65,66,67,68]. Experimental mass spectrometric, ion-mobility spectrometric, and diffusion parameters provide potentials of ion-molecule interaction, thus allowing for the calculation of ion-ion recombination coefficients, average ionic energy, rate of dispersion of ions, electric discharges, different atmospheric phenomena, and more [63]. 

Complementary employment in ion mobility spectrometry, mass spectrometry, and diffusion extracts the so-called collision cross-section of the analyte [63,71,72,73,74,75,76,77,78]. The parameter can be obtained theoretically via high-accuracy methods of computational quantum chemistry using static approaches and molecular dynamics [71,72,73,74,75,76,77,78]. Since data on collision cross-sections provide 3D molecular structures of analytes, experimental MS and ion mobility 3D molecular structures and properties of molecules are correlated with theoretical ones [61,62,71,72,73,74,75,76,77,78,79,80].

Looking at soft-ionization MS methods, electrospray ionization (ESI) and matrix-assisted laser/desorption ionization (MALDI) ones (ESI- and MALDI-MS) are used in many subfields of chemistry, biochemistry, and biology [81,82,83,84,85,86,87,88,89,90,91,92,93,94]. The former method generates analyte molecular-radical and protomer from solution without perturbing specific noncovalent interactions of molecular complexes, if any. For this reason, ESI-MS is a well-suited method for the 3D structural analysis of biologically active molecules. It provides elemental composition and stoichiometry of analytes and their molecular complexes during transfer from solution into gas phase [85,86,87]. ESI-MS is characterized by significant reproducibility and ionization efficiency~100% examining biomacromolecules. The transmission efficiency is 96%. Quantification of peptides yields r^2^ = 0.9899_1_–0.9874_7_ [81]. Singly charged lipid cations generated by MALDI-MS have been first reported herein [83].

There are MS applications to many subfields of analytical and environmental chemistry [61,95,96,97], clinical diagnostics [37], petroleum chemistry, laboratory medicine [98], biochemistry [98], medicinal chemistry, drug design and development of new efficacious therapeutics, forensic chemistry [99], investigations for forensic medico-legal purposes [100], pharmacy [98], toxicology [97,101], nuclear forensics [102], food technology [62], agricultural science, geology, archaeology, etc. MS methods for molecular identification, annotation, and quantification are implemented in metabolomics (*m*/*z* 50–1500) [62], proteomics, (neuro)-proteomics, lipidomics; food-omics, steroid-omics [6,7], glycomics [103], pesticide analysis and control [104], genomics, DNA adduct-omics, transcriptomics, lignomics [10], interactomics [105,106], doping control, petrol-omics, isotomics [27,35], and more. 

Clinical trans-omics is an innovative field integrating clinical phenomes with multi-omics approaches [107]. The precision and reliability of omics methods determine their use in clinical precision medicine. Omics-method performances should be traceable to very high-order analytical standards. Therefore, analytical protocols should have defined uncertainty based on quantitative criteria in statistics and chemometrics [40,61,62,108].

Proteomics provide in-depth knowledge of processes in living cells [16,56,57,109]. The first algorithm elaborated for the purposes of automatic assignment of analyte charge states of ions as well as data-processing methods for deconvolution mass spectra of multiply charged proteins has been developed by Mann and co-workers [5]. Due to limitations in space for this Editorial, it is unable to highlight all contributions devoted to developing algorithms and software for the data processing of MS measurands [110]. 

Metabolomics uses omics methods based on hyphenated instrumentation of chromatography coupled to mass spectrometry [111,112,113] and examines small-molecular metabolites of cellular metabolism [105,106]. It provides insight into biochemical reactions and a comprehensive understanding of the real-time (mechanistic) processes of cells/tissues at the moment of sampling. Due to the high complexity of biological samples, metabolomics methods performances are relative quantitative ones (*r*^2^ = 0.99) [4,114,115]. 

Genetics and transcriptomics answer the following question: What is a cell or tissue capable of doing [105,116]? 

Mass spectrometry determines molecular sequences and modifications, thus addressing many questions about biological processes in vivo. Therefore, it provides crucial knowledge of the relationship between molecular structure and biological function both in vitro and in vivo [117,118]. It also allows us to understand the in-depth neurobiological reactions of neural circuits and cells. First, MALDI-MSI application to clinical diagnostics has been proposed by Caprioli [119,120]. 

The implementation of biochemical methods and mass spectrometry in forensic medico-legal investigations highlights the crucial advantages of analytical mass spectrometry as an objective approach [100].

Lignomics applies MS omics-methods to quantify oligomers of lignin and its derivatives [10].

In addition, the molecules have many molecular isotopologies [27,34,35], showing variation of number of isotopomers. In analyte sample there is concentration of the isotopologically different atoms, perspective, molecular structures of single analyte. It is called sample’s isotome [35]. It encodes data on sample physical and chemical history. Mass spectrometric collisional fragmentation reactions and FTICR or Orbitrap MS analysers detail on sample’s isotome. 

Beyond omics methods, mass spectrometric applications to biochemistry and biology expand dramatically to (macro)molecular structural analysis or the field of structural biology [14]. The reader of this Editorial, perhaps, may not be aware of fundamental issues regarding the utilization of analytical mass spectrometry for analyzing 3D molecular and electronic structure. It, however, methodologically develops the fields of analytical mass spectrometry and structural analysis. 

In the context of the preceding paragraph, single-crystal X-ray diffraction comprises a major method for determining the 3D structures of biological (macro)molecules [121]. However, for purposes of structural biology, it also requires high amounts of pure analytes, good sample crystal growth, and good scattering properties. Frequently, these requirements are major drawbacks of single-crystal X-ray diffraction and its implementation in research on structural biology. 

Computational quantum chemical methods provide high-accuracy data on analyte 3D molecular and electronic structures as well as geometry parameters. Molecular dynamics yields time-dependent results from molecular structure and properties under designed experimental conditions. Theoretical thermodynamics, kinetics, ion mobility, binding affinity, diffusion, catalytic activity, and more parameters allow us to determine the 3D molecular conformation of molecules. The enzyme inactivation reaction step of a biochemical reaction is also obtained. 

Therefore, computational quantum chemistry often overcomes the need to crystallize high-quality single crystals of biologically active (macro)molecules. 

Instrumental methods detailing biochemical reactions and molecular structure include nuclear magnetic resonance, Raman spectroscopy, circular dichroism, and more. However, they often show drawbacks to a broad implementation into biochemistry and structural biology. For instance, nuclear magnetic resonance experiments can be limited to describing the structural differences of biologically active compounds in a small number of sequences [122]. 

Enormous contributions to developing mass spectrometry as a robust instrumental method for chemical analysis have positioned it in the 21st century as analytical instrumentation, having high versatility to identify and quantify biological (macro)molecules and biochemical reactions in vitro and in vivo [61,62,71,72,73,74,75,76,77,78,79,80]. 

Despite this, little attention is focused on the mass spectrometric capability of obtaining exact 3D molecular and electronic structural data using complementary MS measurands and quantum chemical data [61,62,71,72,73,74,75,76,77,78,79,80]. However, proposed candidate structures are often isomeric and have complex electronic effects: tautomeris, isotopomers, protomers, and more. Due to these reasons, accurately determining molecular structure among a set of candidate structures still represents a challenging research task of both the experimental instrumental and theoretical computational methods, even when employing MS instrumentation showing superior method performances or high-accuracy computational quantum chemistry tools [61,62,71,72,73,74,75,76,77,78,79,80]. 

Moreover, mass spectrometric methods utilizing techniques of isotope labeling analytes and H/D exchange yield complete analytical data on the structural consequences of biomacromolecules and the activation biochemical reactions of enzymes. Knowledge develops crucially in the field of biochemistry [122]. 

Methodological developments in exact mass spectrometric methods for 3D structural analysis based on stochastic dynamics approaches to data processing of measurands are also highlighted in the Special Issue [40,61,62].

As previously mentioned, the theme of this Special Issue lies in multi-disciplinary research fields encompassing areas of analytical mass spectrometry, analytical chemistry, and chemometrics, among others, and their application to a broad spectrum of research fields in analytical science. Looking at the content of this Special Issue, it is immediately clear to the reader that among all the theoretical and experimental approaches to mass spectrometry and their applications to numerous multi-disciplinary research fields, it addresses only a few of them. 

However, it provides innovative developments in the fields sketched above for purposes of mass spectrometric-based quantitation and structural analysis of biologically active analytes and samples both in vitro and in vivo, thus highlighting the field of biochemistry. The guest editor, editors, reviewers, and authors were motivated to provide novelty to these scientific fields for researchers and academics working in different disciplines who place their research efforts and innovations into a broader application perspective for fundamental science and industry.

As the guest editor of the Special Issue, I would like to thank all authors who contributed their research and review articles, were devoted to the high quality of their innovative and exciting scientific developments and achievements, and collaborated in publishing them. 

I would like to thank the editors and reviewers for their invaluable and creative recommendations, comments, and remarks, who contributed significantly to the quality of papers and the larger-than-usual review task.
