# Correction: Ivanova, B. Special Issue with Research Topics on “Recent Analysis and Applications of Mass Spectra on Biochemistry”. *Int. J. Mol. Sci.* 2024, *25*, 1995

**DOI:** 10.3390/ijms26073265

**Published:** 2025-04-01

**Authors:** Bojidarka Ivanova

**Affiliations:** Independent Researcher, 44139 Dortmund, Germany; bojidarka.ivanova@yahoo.com or b_ivanova@web.de

Following a request from the University (Dortmund University), a previous affiliate of the Guest Editor (Bojidarka Ivanova) in the published publication [1] has been updated. The authors state that the scientific conclusions are unaffected. This correction was approved by the Academic Editor. The original publication has also been updated.
